# Comparison of in-hospital outcomes after coronary artery bypass graft surgery in elders and younger patients: a multicenter retrospective study

**DOI:** 10.1186/s13019-023-02163-y

**Published:** 2023-02-01

**Authors:** Ren-Jian-Zhi Zhang, Xin-Yi Yu, Jing Wang, Jian Lv, Ming-Huan Yu, Li Wang, Zhi-Gang Liu

**Affiliations:** 1 Department of Cardiovascular Surgery, TEDA International Cardiovascular Hospital, Chinese Academy of Medical Sciences and Peking Union Medical College, 61, Third Avenue, TEDA, Tianjin, China; 2 Department of Cardiovascular Surgery, The First Affiliated Hospital of Zhengzhou University, Zhengzhou, China; 3 Department of Cardiovascular Surgery, Nanyang Central Hospital, Nanyang, China

**Keywords:** Outcomes, Coronary artery bypass graft surgery, Age

## Abstract

**Objectives:**

We aimed to identify in-hospital outcomes in young (≤ 65 years) and old (> 65 years) patients after coronary artery bypass grafting (CABG) by analyzing the effect of age on adverse events after on-pump or off-pump CABG.

**Methods:**

Patients older than 65 years were defined as older patients and others were defined as younger patients. The qualitative data were compared by chi-square or Fisher's exact tests. The quantitative data were compared by the two-sample independent t-test or Mann–Whitney U test. Multifactor binary logistic regression was used to control for confounders and to investigate the effect of age on dichotomous outcome variables such as death.

**Results:**

In the on-pump CABG population, the postoperative in-hospital mortality, the incidence of postoperative symptomatic cerebral infarction (POSCI) and postoperative atrial fibrillation (POAF) was higher in older patients than in younger patients (*P* value < 0.05), and age > 65 years was associated with postoperative in-hospital mortality (*OR* = 2.370, *P* value = 0.031), POSCI (*OR* = 5.033, *P* value = 0.013), and POAF (*OR* = 1.499, *P* value < 0.001). In the off-pump CABG population, the incidence of POAF was higher in older patients than in younger patients (*P* value < 0.05), and age > 65 years was associated with POAF (*OR* = 1.392, *P* value = 0.011).

**Conclusion:**

In-hospital outcomes after CABG are strongly influenced by age. In on-pump CABG, the risk of postoperative death, POSCI, and POAF was higher in older patients, and in off-pump CABG, the risk of POAF was higher in older patients.

## Introduction

Coronary artery disease (CAD) is a prominent cause of morbidity and mortality across the world [1]. Currently, the best method for revascularization of multiple coronary stenoses or severe left main lesions is coronary artery bypass grafting (CABG), which can greatly improve patient prognosis and quality of life. Depending on whether cardiopulmonary bypass (CPB) is employed, CABG is classified as on-pump CABG or off-pump CABG. Comparison of in-hospital outcomes or long-term outcomes between the two procedures has been reported in some papers [2, 3]. The results of the studies from different medical institutions were inconsistent, and none of them revealed differences in postoperative outcomes between patients in different age groups with on-pump CABG or off-pump CABG.

Age is associated with multiple adverse events after CABG [4]. A comparison of postoperative outcomes of CABG in different age groups with different procedures is therefore necessary. Multi-center retrospective studies help reduce bias and improve the credibility of results. In this study, CABG patients from three medical centers in different parts of China, of different sizes and levels, were selected as the research population. The differences in postoperative in-hospital outcomes of CABG in younger and older patients with different surgical approaches were examined. The purpose of this study is to determine in-hospital outcomes in patients of different ages (≤ 65 years and > 65 years) after CABG and provide prevention guidance to clinical practice.

## Materials and methods

### Research population

This was a retrospective multicenter research. All data from national triple A, first-class medical center in China with extensive experience in CABG. And all operations are performed by cardiac surgeons with more than 10 years of experience in CABG. Data was collected from (1) all patients admitted to TEDA International Cardiovascular Hospital and got CABG from September 2020 to December 2021; (2) all patients admitted to the First Affiliated Hospital of Zhengzhou University and got CABG from January 2020 to December 2021; and (3) all patients admitted to Nanyang Central Hospital and got CABG from January 2020 to December 2021.

The inclusion criteria were: (1) patients over the age of 18; (2) patients who were admitted to the hospital for the first time and had CABG without prior cardiac surgery; and (3) the bypass vessels were the internal mammary artery and/or great saphenous vein. Patients with aortic dissection or aortic valve replacement will be excluded.

## Research data

Patients over 65 years old were classified as elderly, while others were classified as youthful. Preoperative characteristics included gender, age, body mass index (BMI), smoking, diabetes, hypertension, and prior percutaneous coronary intervention (PCI). Diabetes mellitus is defined as previously diagnosed with diabetes mellitus or newly diagnosed with diabetes mellitus on hospital admission: (1) typical symptoms of diabetes (such as polyuria and polydipsia) + random venous blood glucose ≥ 11.1 mmol/L. (2) Fasting venous blood glucose ≥ 7.0 mmol/L. (3) Two-hour intravenous glucose ≥ 11.1 mmol/L during oral glucose tolerance test. Hypertension is defined as previously diagnosed with hypertension or newly diagnosed with hypertension on hospital admission: Systolic blood pressure ≥ 140 mmHg and/or diastolic blood pressure ≥ 90 mmHg on 3 non-same day measurements without anti-hypertensive medication. Medication usage before surgery included β-blockers and statins. All laboratory indications, including PaO_2_, PaCO_2_, pH and lactate of arterial blood gas, leukocyte count, erythrocyte count, neutrophil-to-lymphocyte ratio (NLR), hemoglobin, platelet count, albumin-to-globulin ratio (A/G), low-density lipoprotein to high-density lipoprotein ratio (LDL/HDL), aspartate aminotransferase to alanine aminotransferase ratio (AST/ALT), creatinine, and glucose. Other preoperative indicators included the presence of left main disease, left ventricular ejection fraction (LVEF), ventricular aneurysm, left ventricular end-diastolic diameter (LVEDD), and left atrial diameter (LAD). All the above preoperative indicators were the last results before surgery. CPB usage, CPB duration, and intracardiac operation (refers to a surgical procedure that opens the atrium or septum in conjunction with CABG, such as valve replacement) were intraoperative characteristics. The initial 24-h volume status was one of the postoperative characteristics (i.e., the difference between the total liquid in and liquid out over a 24 h).

### In-hospital outcomes

The primary outcome was postoperative in-hospital death. The secondary outcomes include postoperative myocardial infarction (POMI), postoperative symptomatic cerebral infarction (POSCI), postoperative pulmonary embolism (POPE), postoperative atrial fibrillation (POAF), the duration of postoperative mechanical ventilation (POMV), and the length of stay (LOS) in hospital. The definitions or diagnoses of the above in-hospital outcomes are shown in Table 1.

**Table 1:** The definitions or diagnoses of in-hospital outcomes

Outcomes	Definition/diagnosis
Post-operative death	The post-operative death is defined as in-hospital mortality due to a variety of reasons following surgery
POMI	POMI is defined by the satisfaction of three of the criteria below: (1) Abnormal changes in ECG waveforms, such as ST-segment elevation, pathological Q waves, etc.; (2) troponin and myocardial enzyme levels were greater than the upper limit of normal and tended to rise. (3) Typical clinical symptoms, such as sudden onset of severe and persistent retrosternal or precordial crushing pain that is not relieved by rest or nitroglycerin
POSCI	POSCI is diagnosed by the satisfaction of four of the criteria below: (1) the existence of cerebrovascular disease risk factors; (2) the presence of unrelieved neurological impairments indicators; (3) cranial CT to rule out bleeding; (4) cranial MR to confirm cerebral infarction
POPE	POPE was diagnosed by computed tomography pulmonary angiog-raphy
POAF	POAF is defined by the satisfaction of three of the criteria below: (1) accelerated heart rate; (2) the electrocardiogram waveform indicated that P wave disappeared and was replaced by the irregular f wave; (3) the onset lasted more than 10 min
The duration of POMV (hour)	The duration is defined as the period from the end of surgery to the recovery of spontaneous breathing and extubated from tracheal intubation
LOS in hospital (day)	LOS refers to the total time between admission and discharge from the hospital

*POMI* postoperative myocardial infarction, *POSCI* postoperative symptomatic cerebral infarction, *POPE* postoperative pulmonary embolism, *POAF* postoperative atrial fibrillation, *POMV* postoperative mechanical ventilation, *LOS* length of stay

### Ethics and research quality

The authors are accountable for all aspects of the work in ensuring that questions related to the accuracy or integrity of any part of the work are appropriately investigated and resolved. The study was conducted in accordance with the Declaration of Helsinki (as revised in 2013). The study was approved by the Ethics Committee of TEDA International Cardiovascular Hospital (Internal Review Board) and individual consent for this retrospective analysis was waived. Surgeons are well trained at each medical institution, and surgical operations and CPB are carried out in accordance with standards and under stringent quality supervision. Epidata 3.1 was used to enter all of the data for the research. Following the completion of data input, a thorough review was undertaken to examine the original data and correct any mistakes.

### Statistical analysis

R software (ver 4.1.2, USA) was used to perform data analysis. The qualitative data were expressed as frequencies or ratios and compared by chi-square or Fisher's exact tests. The quantitative data were tested for normality using the Shapiro–Wilk method. Normally distributed quantitative data were expressed as mean ± standard deviation and compared by the two-sample independent t-test. Non-parametric quantitative data were expressed as the median and interquartile range (IQR) and compared by the Mann–Whitney U test. Multifactor binary logistic regression was used to control for confounders and to investigate the effect of age on dichotomous outcome variables such as death (for non-parametric confounders, the natural logarithm was taken to improve normality). Statistical significance is indicated by a *P* value < 0.05.

## Results

### Baseline characteristics

**Fig. 1:**
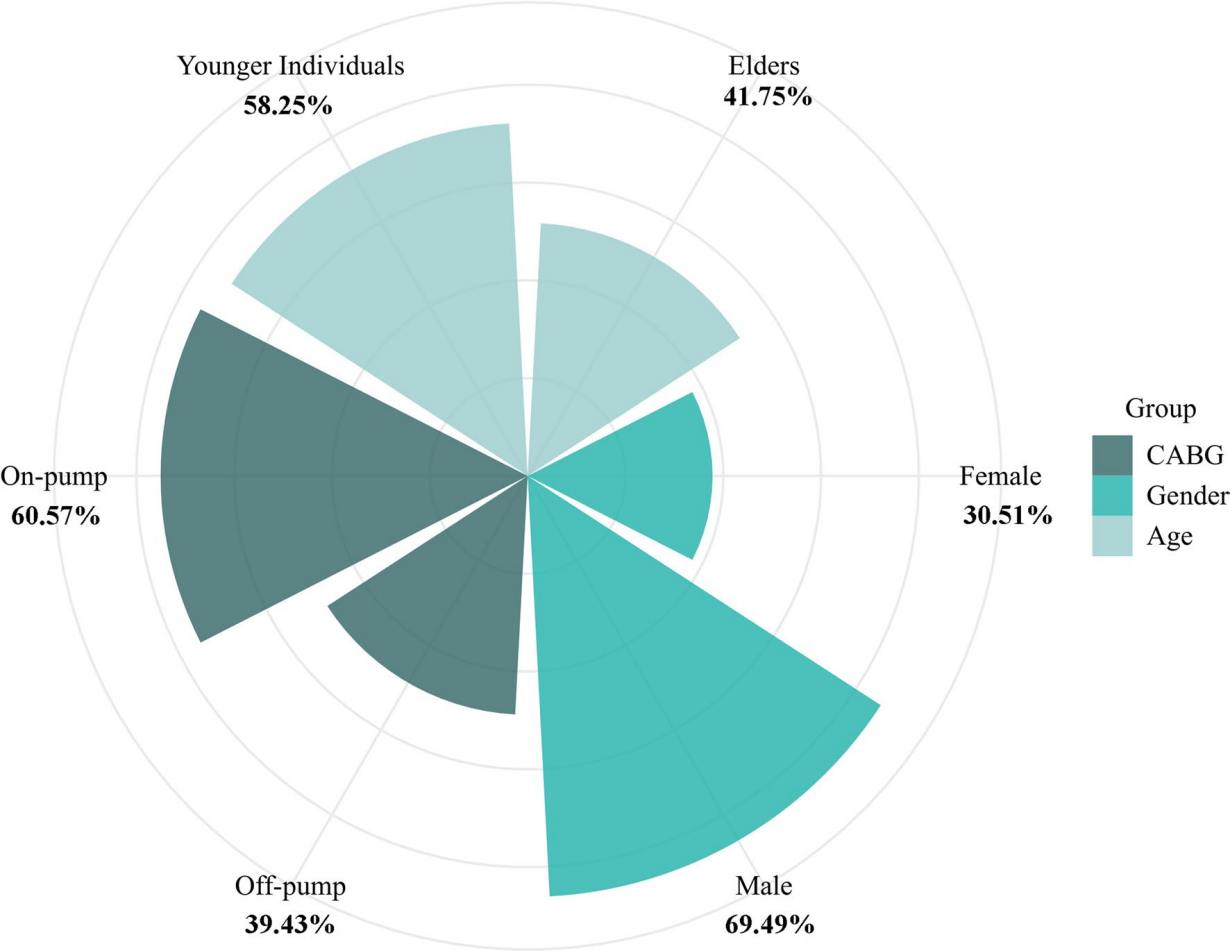
The proportions of surgical modalities, gender, and age in the study population

In this study, 3097 patients were included from 3 medical centers, and the composition ratios of surgical modality, gender, and age are shown in Fig. 1. On-pump CABG was performed on 1876 patients, while off-pump CABG was performed on 1221 patients. Male patients numbered 2152 and female patients numbered 945. There were 1293 elderly patients over 65 years old (70.57 ± 3.72 years old) and 1804 younger patients (57.32 ± 6.57 years old). In Table 2, perioperative baseline data is compared for younger and older groups. In the on-pump CABG population, younger patients ≤ 65 years of age had a higher BMI, a greater percentage of male patients, a greater percentage of smokers, a greater percentage of diabetic patients, and a greater percentage of left main lesions compared with older patients. A lower percentage of patients had a history of hypertension compared with the older group (*P* value < 0.05). As for the off-pump CABG population, the proportion of male patients and the proportion of smokers were higher in the younger group (*P* value < 0.05).

**Table 2:** Comparison of elderly and younger patients at perioperative baseline

Indicators	On-pump CABG (n = 1876)			Off-pump CABG (n = 1221)		
	Younger patients (*n* = 1076)	Elderly patients (*n* = 800)	*P* value	Younger patients (*n* = 728)	Elderly patients (*n* = 493)	*P* value
Gender			< 0.001			< 0.001
Female	281 (26.12%)	292 (36.50%)		183 (25.14%)	189 (38.34%)	
Male	795 (73.88%)	508 (63.50%)		545 (74.86%)	304 (61.66%)	
BMI (kg/m^2^)	25.82 (23.88, 27.92)	25.07 (23.17, 27.34)	< 0.001	26.01 (23.83,27.94)	25.66 (23.39,28.05)	0.272
Smoking			< 0.001			0.002
No	597 (55.48%)	552 (69.00%)		469 (64.42%)	360 (73.02%)	
Yes	479 (44.52%)	248 (31.00%)		259 (35.58%)	133 (26.98%)	
Diabetes			0.024			0.093
No	639 (59.39%)	516 (64.50%)		513 (70.47%)	325 (65.92%)	
Yes	437 (40.61%)	284 (35.50%)		215 (29.53%)	168 (34.08%)	
Hypertension			0.004			0.418
No	344 (31.97%)	207 (25.87%)		275 (37.77%)	175 (35.50%)	
Yes	732 (68.03%)	593 (74.12%)		453 (62.23%)	318 (64.50%)	
PCI			0.628			0.077
No	881 (81.88%)	648 (81.00%)		627 (86.13%)	442 (89.66%)	
Yes	195 (18.12%)	152 (19.00%)		101 (13.87%)	51 (10.34%)	
Pre-operative β-blockers medication			0.827			0.951
No	267 (24.81%)	195 (24.38%)		85 (11.68%)	57 (11.56%)	
Yes	809 (75.19%)	605 (75.62%)		643 (88.32%)	436 (88.44%)	
Pre-operative statin medication			0.009			0.578
No	63 (5.86%)	72 (9.00%)		21 (2.88%)	17 (3.45%)	
Yes	1013 (94.14%)	728 (91.00%)		707 (97.12%)	476 (96.55%)	
pH	7.43 (7.39, 7.47)	7.43 (7.39, 7.46)	0.075	7.42 (7.39, 7.46)	7.43 (7.39, 7.46)	0.742
PaO_2_ (mmHg)	130.00 (102.00, 166.00)	132.00 (106.00, 167.00)	0.238	96.00 (81.00, 132.75)	106.00 (85.00, 148.50)	0.001
PaCO_2_ (mmHg)	34.00 (30.00, 38.00)	34.00 (31.00, 39.00)	0.080	38.00 (33.00, 42.00)	38.00 (32.00, 41.00)	0.423
Lactate (mmol/L)	1.00 (0.80, 1.50)	1.10 (0.80, 1.60)	0.106	1.40 (0.90, 1.90)	1.20 (0.90, 1.90)	0.047
WBC (10^9^/L)	6.40 (5.40, 7.50)	6.30 (5.30, 7.50)	0.456	6.20 (5.30, 7.37)	6.19 (5.40, 7.20)	0.538
RBC (10^12^/L)	4.40 (4.10, 4.70)	4.40 (4.00, 4.80)	0.578	4.50 (4.15, 4.90)	4.44 (4.16, 4.81)	0.482
Hb (g/L)	135.00 (124.00, 147.00)	136.00 (123.00, 147.00)	0.872	134.00 (122.00, 143.00)	133.00 (121.00, 142.00)	0.715
NLR	2.13 (1.57, 2.84)	2.07 (1.53, 2.75)	0.131	1.88 (1.46, 2.60)	1.93 (1.46, 2.57)	0.766
PLT (10^9^/L)	215.00 (179.00,255.00)	217.00 (182.00, 255.00)	0.711	178.50 (154.00, 224.00)	187.00 (157.00, 237.00)	0.004
A/G	1.61 (1.45, 1.79)	1.61 (1.46, 1.81)	0.532	1.57 (1.40, 1.75)	1.54 (1.39, 1.73)	0.291
LDL/HDL	2.65 (2.07, 3.36)	2.63 (2.03, 3.33)	0.384	2.52 (1.99, 3.24)	2.49 (1.94, 3.04)	0.317
AST/ALT	0.89 (0.68, 1.14)	0.91 (0.70, 1.17)	0.529	0.96 (0.76, 1.20)	0.90 (0.73, 1.14)	0.020
Creatinine (μmol/L)	69.00 (58.00, 81.00)	69.00 (58.25, 81.00)	0.997	73.00 (62.00, 87.00)	72.00 (61.00, 85.00)	0.137
Glucose (mmol/L)	5.70 (5.10, 6.80)	5.70 (5.10, 6.70)	0.575	5.72 (5.10, 6.74)	5.90 (5.20, 6.97)	0.070
Left main stem lesion			0.005			0.238
No	771 (71.65%)	619 (77.38%)		474 (65.11%)	337 (68.36%)	
Yes	305 (28.35%)	181 (22.62%)		254 (34.89%)	156 (31.64%)	
LVEF (%)	61.00 (55.00, 66.00)	61.00 (55.25, 65.00)	0.806	60.00 (57.00, 63.00)	60.00 (57.00, 64.00)	0.014
LVEDD (mm)	48.00 (45.00, 52.00)	48.00 (45.00, 52.00)	0.474	47.00 (44.00, 49.00)	47.00 (45.00, 50.00)	0.023
LAD (mm)	37.00 (35.00, 40.00)	38.00 (35.00, 40.00)	0.920	35.00 (33.00, 38.00)	36.00 (33.00, 39.00)	0.038
Intracardiac Operation			0.141	/	/	/
No	945 (87.83%)	684 (85.50%)				
Yes	131 (12.17%)	116 (14.50%)				
CPBtime (min)	95.00 (79.00, 120.00)	95.00 (77.00, 121.00)	0.912	/	/	/
Initial 24-h volume status (mL)	− 312.00 (− 759.75, 107.25)	− 290.00 (− 716.50, 95.00)	0.534	− 145.00 (− 481.50, 184.00)	− 145.00 (− 563.00, 186.00)	0.394

The qualitative data were compared by chi-square or Fisher's exact tests. Normally distributed quantitative data were compared by the two-sample independent t-test. Non-parametric quantitative data were compared by the Mann–Whitney U test

*BMI* body mass index, *PCI* percutaneous coronary intervention, *PaO*_*2*_ arterial partial pressure of oxygen, *PaCO*_*2*_ arterial partial pressure of carbon dioxide, *WBC* leukocyte count, *RBC* erythrocyte count, *Hb* hemoglobin, *NLR* neutrophil-to-lymphocyte ratio, *PLT* platelet count, *A/G* albumin-to-globulin ratio, *LDL/HDL* low-density lipoprotein to high-density lipoprotein ratio, *AST/ALT* aspartate aminotransferase to alanine aminotransferase ratio, *LVEF* left ventricular ejection fraction, *LVEDD* left ventricular end-diastolic diameter, *LAD* left atrial diameter, *CPBtime* the duration of cardiopulmonary bypass

### In-hospital outcomes

**Fig. 2:**
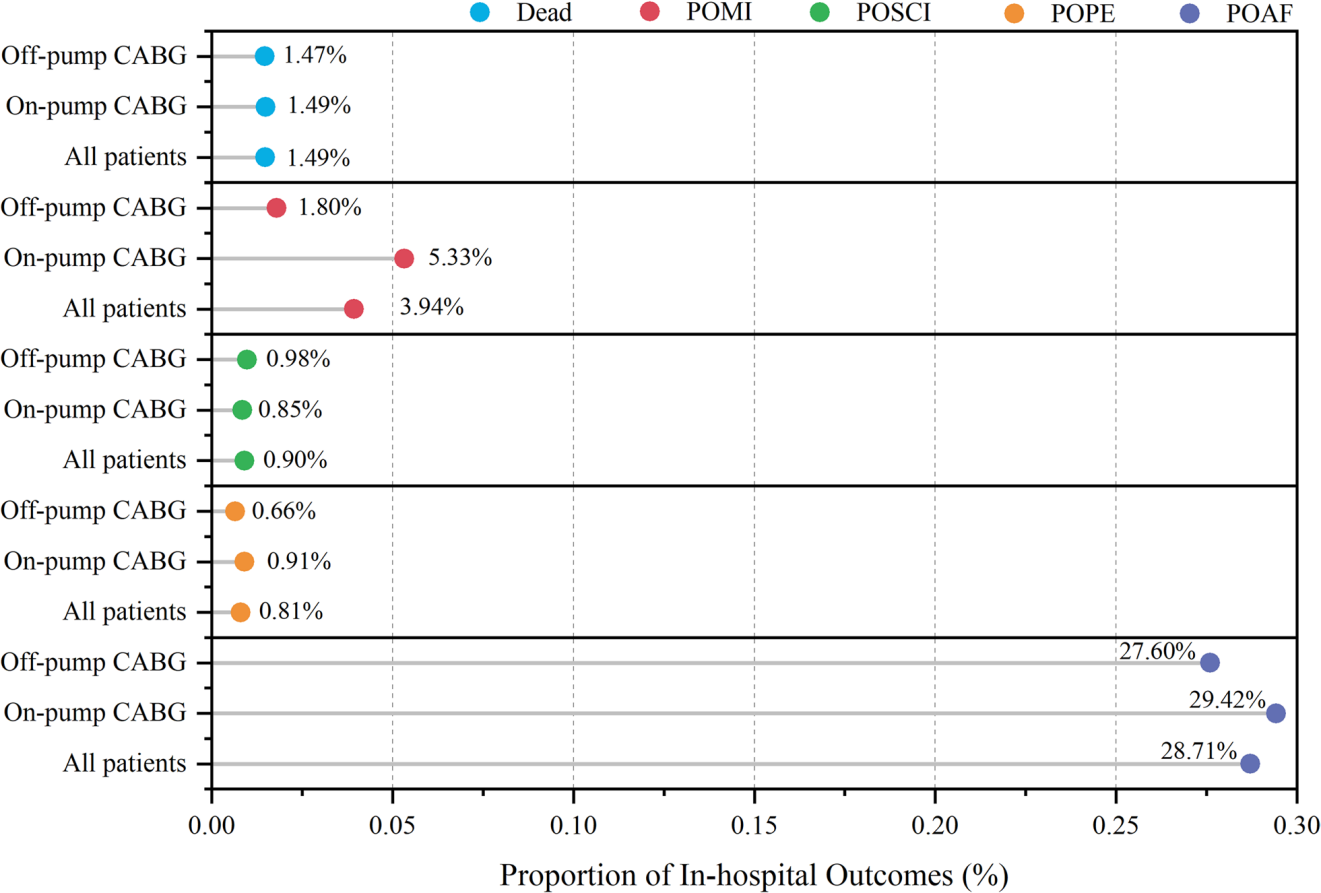
Incidence of in-hospital outcomes for all patients, the on-pump CABG population, and the off-pump CABG population. *POMI* postoperative myocardial infarction, *POSCI* postoperative symptomatic cerebral infarction, *POPE* postoperative pulmonary embolism, *POAF* postoperative atrial fibrillation

Figure 2 shows the incidence of primary and secondary outcomes in overall study population, on-pump CABG population, and off-pump CABG population. The median duration of POMV and LOS in hospital for overall study population was 12.00 (8.00, 16.00) hours and 18.00 (15.00, 23.00) days, respectively. The results of the comparison between ≤ 65 and > 65 years old with on-pump or off-pump CABG are shown in Table 3. In the on-pump CABG population, postoperative mortality, POSCI incidence, and POAF incidence were higher in the elderly group than in the younger group of patients, and LOS in hospital was longer (*P* value < 0.05). In the off-pump CABG population, the incidence of POAF was higher in the older group than in the younger group, and the duration of POMV and LOS in hospital was longer (*P* value < 0.05).

**Table 3:** Comparison of in-hospital outcomes among patients of different ages

Outcomes	On-pump CABG (*n* = 1876)			Off-pmup CABG (*n* = 1221)		
	Younger patients (*n* = 1076)	Elderly patients (*n* = 800)	*P* value	Younger patients (*n* = 728)	Elderly patients (*n* = 493)	*P* value
Post-operative death			0.020			0.723
No	1066 (99.07%)	782 (97.75%)		718 (98.63%)	485 (98.38%)	
Yes	10 (0.93%)	18 (2.25%)		10 (1.37%)	8 (1.62%)	
POSCI			0.002			0.771
No	1073 (99.72%)	787 (98.38%)		720 (98.90%)	489 (99.19%)	
Yes	3 (0.28%)	13 (1.62%)		8 (1.10%)	4 (0.81%)	
POAF			< 0.001			0.009
No	798 (74.16%)	526 (65.75%)		547 (75.14%)	337 (68.36%)	
Yes	278 (25.84%)	274 (34.25%)		181 (24.86%)	156 (31.64%)	
POMI			0.266			0.699
No	1024 (95.17%)	752 (94.00%)		714 (98.08%)	485 (98.38%)	
Yes	52 (4.83%)	48 (6.00%)		14 (1.92%)	8 (1.62%)	
POPE			0.388			> 0.999
No	1068 (99.26%)	791 (98.88%)		723 (99.31%)	490 (99.39%)	
Yes	8 (0.74%)	9 (1.12%)		5 (0.69%)	3 (0.61%)	
LOS in hospital (day)	17.00 (14.00, 21.00)	18.00 (15.00, 23.00)	< 0.001	19.00 (15.25, 25.00)	21.00 (16.00, 26.00)	0.003
The duration of POMV (hour)	11.00 (8.00, 17.00)	11.50 (8.00, 17.00)	0.565	12.00 (9.00, 15.00)	12.00 (10.00, 15.00)	0.001

The qualitative data were compared by chi-square or Fisher’s exact tests. Non-parametric quantitative data were compared by the Mann–Whitney U test

*POMI* postoperative myocardial infarction, *POSCI* postoperative symptomatic cerebral infarction, *POPE* postoperative pulmonary embolism, *POAF* postoperative atrial fibrillation, *POMV* postoperative mechanical ventilation, *LOS* length of stay

### The relationship between advanced age and post-operative outcomes

A multifactorial binary logistic regression was used to examine the effect of age on categorical outcome indicators after controlling for confounding factors. A comparison between groups was used to determine confounding factors. In the on-pump CABG population, confounders associated with postoperative death were preoperative lactate level (*P* value = 0.012), A/G (*P* value = 0.042), and CPB duration (*P* value < 0.001); confounders associated with POSCI were preoperative statin administration (*P* value = 0.004), PaO_2_ level (*P* value = 0.025), and intraoperative combined intracardiac operation (*P* value = 0.049); the confounding factor related to POAF was preoperative leukocyte count (*P* value = 0.011). After adjusting for confounders, age > 65 years associated with on-pump CABG postoperative death (*OR* = 2.370, 95%*CI* 1.082–5.194, *P* value = 0.031), POSCI (*OR* = 5.033, 95%*CI* 1.411–17.952, *P* value = 0.013), and POAF (*OR* = 1.499, 95%*CI* 1.227–1.832, *P* value < 0.001). The confounding factors associated with POAF in the off-pump CABG population were history of hypertension (*P* value = 0.034) and preoperative left atrial internal diameter (*P* value = 0.006). After adjusting for confounders, age > 65 years associated with POAF of off-pump CABG (*OR* = 1.392, 95%*CI* 1.080–1.794, *P* value = 0.011).

## Discussion

Age is not only a risk factor for CAD, but is also considered to be a factor affecting postoperative outcomes in CABG [5, 6]. The study population was selected from 3 different medical centers in China, each with a varying surgical volume. In the study population, 41.75% of patients were elderly (> 65 years), 69.49% were males, and the primary surgical procedure was on-pump CABG (60.57%). In the overall study sample, the in-hospital all-cause mortality rate after CABG was 1.49%, and the incidence of POMI, POSCI, POPE, and POAF was 3.94%, 0.90%, 0.81%, and 28.71%, respectively; the median duration of POMV and LOS in hospital was 12 h and 18 days, respectively. The results are similar to those of some previous studies [7–9]. It has been suggested that females have a higher risk of adverse cardiovascular events and death after CABG [10, 11]. However, gender was not a confounding factor during the data analysis of this study. The high proportion of male patients may have reduced the effect of gender on the statistical results. Mid-term survival and cardiovascular adverse event trends for on-pump CABG and off-pump CABG have been suggested to be comparable [12, 13]. But on-pump CABG and off-pump CABG may differ in serum cytokine and chemokine levels and oxidative stress status, which may affect postoperative outcomes [14, 15]. Therefore, this study differentiated the surgical procedures.

Although the underlying mechanism of age and atrial fibrillation (AF) remains unclear, it has been recognized as one of the major risk factors for POAF after CABG [16–18]. In the case of POAF after CABG, the patient's cardiac function can be negatively affected, leading to circulatory instability and myocardial ischemia, which then affects the patient's postoperative recovery, resulting in longer ICU stays, longer hospital stays, increased medical costs, increased IABP requirements, and even death [19–21]. According to this study, POAF was a common complication both in on-pump and off-pump CABG, and the incidence of POAF was about 25% in younger patients (≤ 65 years old), and about 30% in older patients (> 65 years old). Logistic regression analysis showed that in the on-pump population, the risk of POAF was 1.499 times higher in older patients > 65 years of age than in younger patients; in the off-pump population, it was 1.392 times higher in older patients > 65 years of age than in younger patients. According to Roberts et al. biological aging plays an important role in the occurrence and development of incident AF [22]. In addition to biological aging, we suggest that different age groups have different stress states during surgery and different levels of postoperative inflammatory factors/cytokines, which result in the different incidence of POAF.

Cerebrovascular complications after CABG are likewise associated with higher rates of in-hospital mortality, longer lengths of hospital stay, and higher total hospitalization cost [23]. Ito, Mohamed, and Loberman et al. concluded that age was one of the most significant predictors of cerebrovascular complications following CABG [23–25]. Because this study was a retrospective analysis and cranial CT or MRI was not performed for all patients after CABG, the rate of CI may have been considerably underestimated. Therefore, this study focused only on POSCI incidence. According to the results of this study, the incidence of POSCI after on-pump CABG was 0.28% and 1.62% in younger and older patients, respectively, and the incidence of POSCI after off-pump CABG was 1.10% and 0.81%. In the population of patients undergoing on-pump CABG, the incidence of POSCI was higher in elderly patients > 65 years old than in younger patients (*P* value < 0.05), and logistic regression analysis indicated that the risk of POSCI was 5.033 times higher in older patients > 65 years old than in younger patients. However, in the off-pump CABG population, there was no difference in the incidence of POSCI between the different age groups (*P* value > 0.05). Naito et al. [26] suggested that in patients with increased risk of perioperative stroke, aortic manipulation including the use of cardiopulmonary bypass or partial clamping for central anastomoses is associated with higher rates of postoperative neurological complications. For elderly patients > 65 years of age, the trend in the incidence of POSCI after off-pump CABG versus on-pump CABG shown in this study was generally consistent with previous studies [27, 28]. The incidence of POSCI after off-pump CABG was comparable in elderly and younger patients. Off-pump CABG may reduce the risk of postoperative CI and may be safe and effective for preventing postoperative cardiovascular and cerebrovascular accidents in elderly patients.

In elderly patients who undergo CABG, mortality tends to increase [29]. The results of this study showed that in-hospital all-cause mortality after on-pump CABG was 0.93% and 2.25% in younger and older patients, respectively, and 1.37% and 1.62% after off-pump CABG. In the on-pump CABG population, postoperative all-cause mortality was higher in older patients > 65 years than in the younger group (*P* value < 0.05). The risk of postoperative in-hospital death was 2.370 times higher in patients > 65 years of age than in younger patients, according to logistic regression analysis. There were no differences in postoperative mortality between the different age groups in the off-pump CABG population (*P* value > 0.05). Senior-aged patients commonly have a more severe disease status combined with more frequent comorbidities, which may lead to a high risk of mortality [30]. For elderly patients > 65 years of age, the trend in in-hospital mortality after off-pump CABG versus on-pump CABG was generally consistent with previous studies. According to Ohira et al., short-term death after off-pump CABG is not related to age [31]. According to Khan et al. off-pump CABG has a lower mortality rate than on-pump CABG in elderly patients, and that using off-pump CABG in elderly patients could reduce the burden on healthcare providers [32].

In addition to the above in-hospital outcomes, the median LOS in hospital was 18 and 17 days for older and younger patients, respectively, in the on-pump CABG population (*P* value < 0.001), and 21 and 19 days for older and younger patients, respectively, in the off-pump CABG population (*P* value = 0.003). The results of the study showed that LOS in hospital was greater in older patients than in younger patients, regardless of the surgical procedures. In addition, we found that off-pump CABG patients had longer hospital stays than those undergoing on-pump CABG, regardless of the patient's age. The reason for this discrepancy may be related to preoperative status and duration of ventilator use, but further investigation is needed to pinpoint the exact cause.

The study has some limitations. This is an observational study with relatively broad inclusion and exclusion criteria, which allows for a more authentic presentation of the population baseline data. However, this inevitably increases the confounding factors. Considering this is a retrospective study, if patients are selected for the study over a longer period of time, even though the sample size increases, potential confounding factors (i.e., different surgeons, bypass surgery, etc.) affecting the treatment process may result in bias. The study therefore focused on patients from the last 2 years, however this also led to a new problem, namely, a lack of young patients under 45 years of age and senior patients over 80 years of age, which resulted in the failure to complete the analysis of the four age groups of young, middle-aged, elderly, and senior patients (> 80 years old). However, even if only patients who have undergone CABG in the last 2 years are selected, there may be some difference in the details of the operation between hospitals and even between surgeons, which may also produce confounding. In future studies, multicenter prospective studies should be conducted to adjust for confounding factors, as well as refine age groupings to explore the relationship between age and early, intermediate, and long-term outcomes after CABG.

## Conclusion

Patients of advanced age have a higher risk of adverse hospital outcomes after CABG. For on-pump CABG, the risk of postoperative death, risk of POSCI, and risk of POAF were approximately 2, 5, and 1.5 times higher in older patients > 65 years of age than in younger patients, respectively. For off-pump CABG, the risk of POAF was approximately 1.4 times higher in elderly patients > 65 years of age than in younger patients. Moreover, the LOS in the hospital was longer in older patients > 65 years of age regardless of the surgical procedure. Off-pump CABG has fewer adverse outcomes in the short term in older patients and may be more cost-effective. Preoperative risk assessment for CABG according to age is particularly important, and rigorous management of reversible risk factors and individualized treatment will be key to the scientific implementation of the surgical procedure.

## Data Availability

The data that support the findings of this study are available from the corresponding author upon reasonable request.

## Abbreviations

CAD: Coronary artery disease
CABG: Coronary artery bypass grafting
CPB: Cardiopulmonary bypass
BMI: Body mass index
PCI: Percutaneous coronary intervention
NLR: Neutrophil-to-lymphocyte ratio
A/G: Albumin-to-globulin ratio
LDL/HDL: Low-density lipoprotein to high-density lipoprotein ratio
AST/ALT: Aspartate aminotransferase to alanine aminotransferase ratio
LVEF: Left ventricular ejection fraction
LVEDD: Left ventricular end-diastolic diameter
LAD: Left atrial diameter
POMI: Post-operative myocardial infarction
POSCI: Post-operative symptomatic cerebral infarction
POPE: Post-operative pulmonary embolism
POAF: Postoperative atrial fibrillation
POMV: Post-operative mechanical ventilation
LOS: Length of stay
AF: Atrial fibrillation
